# The Perfect Storm: A Case of Ischemic Stroke in the Setting of Thyroid Storm

**DOI:** 10.7759/cureus.7992

**Published:** 2020-05-06

**Authors:** Samantha Snyder, Maria Joseph

**Affiliations:** 1 Internal Medicine, University of Pittsburgh Medical Center Pinnacle, Harrisburg, USA

**Keywords:** thyroid storm, atrial fibrillation, stroke, thyrotoxicosis, management

## Abstract

Mortality in thyroid storm, without appropriate treatment, can rise as high as 100%. Thyroid storm coexisting with ischemic stroke is a rare presentation that further increases the risk of mortality. Early recognition and appropriate management are critical to the prevention of mortality and morbidity. Here, we review the case of a 63-year-old male presenting with new neurological deficits who was found to have thyroid storm; appropriate management of the co-existing conditions are also reviewed.

## Introduction

Thyroid storm is a life-threatening condition due to uncontrolled hyperthyroid state with an overall mortality as high as 10% to 60% [1-3]. It can be precipitated by a variety of events, including infection, surgery, trauma, iodine load, and medication non-compliance [2-6]. In these patients, there are increased rates of cardiomyopathy, cardiovascular disease, and arrhythmias, particularly atrial fibrillation when compared to the general population [1,6,7]. 

Thyroid storm coexisting with ischemic stroke is a rare presentation. In these scenarios, ischemic stroke can either act as the acute illness precipitating the thyroid storm or can be the direct result of the storm. Treatment relies on both appropriate stroke and thyrotoxicosis management. When the conditions co-exist, adjustments in management are necessary to ensure proper care.

## Case presentation

**Figure 1:**
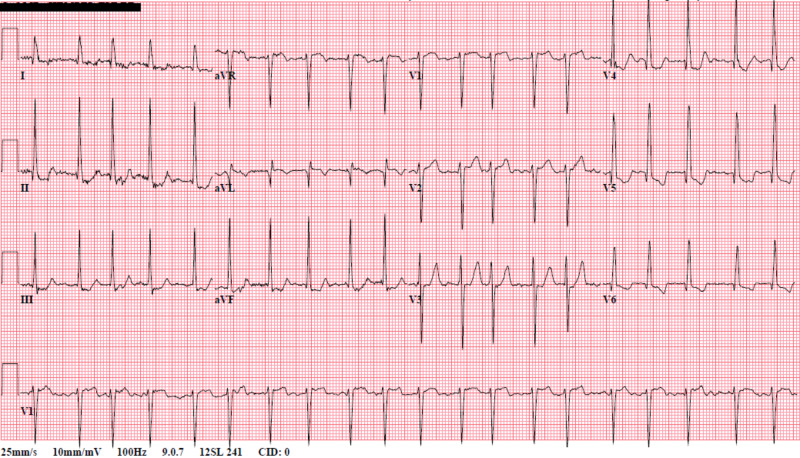
Electrocardiogram showing atrial fibrillation with rapid ventricular rate

**Figure 2:**
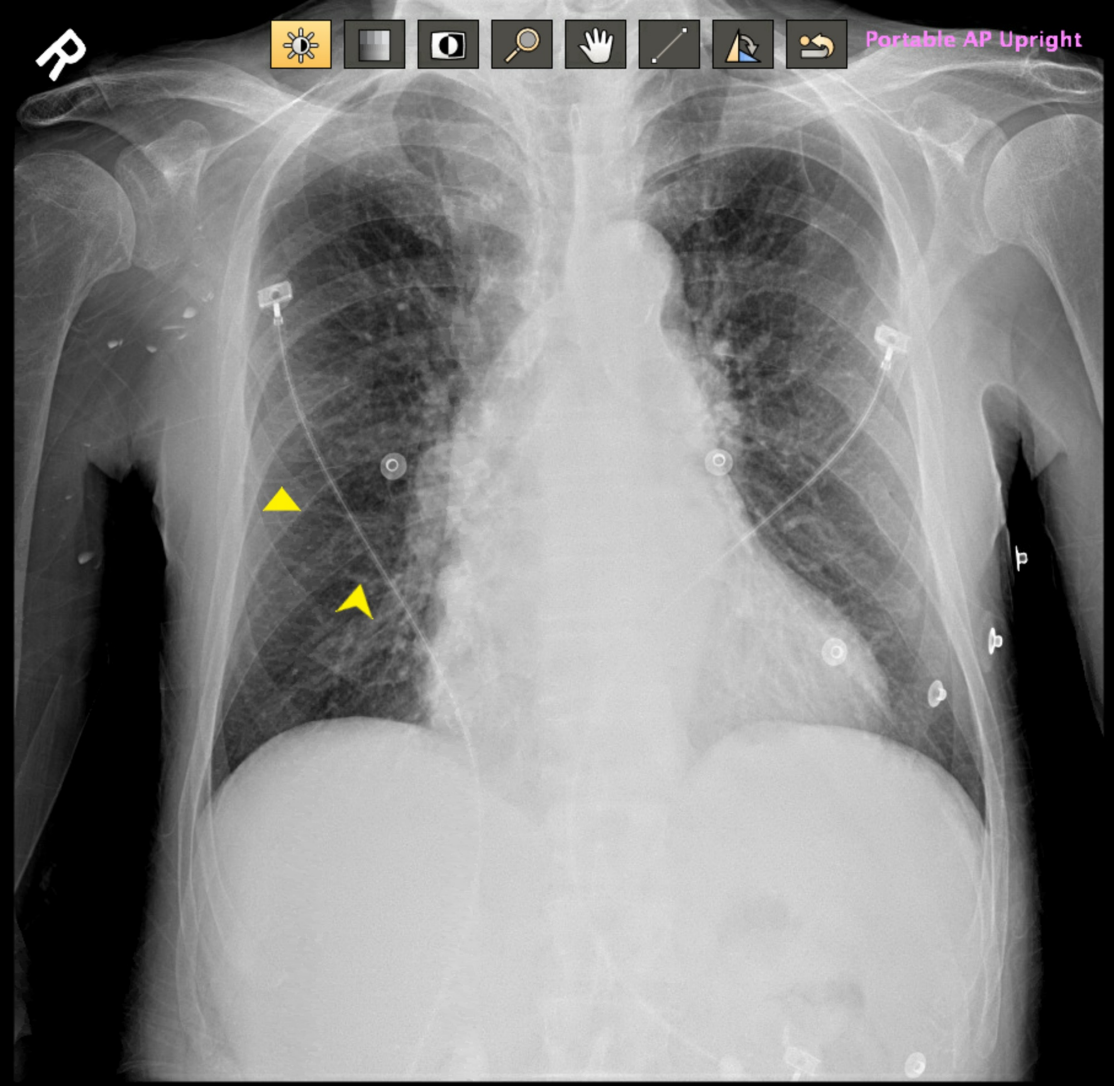
Chest x-ray revealing mild pulmonary edema Yellow arrows marking Kerley B lines suggestive of pulmonary edema

A 63-year-old male with a past medical history of bipolar disorder, alcohol abuse in remission, hypertension, and medical non-compliance presented for evaluation of left-sided weakness and slurred speech. On initial evaluation, the patient was found to be hypertensive (172/101 mmHg) and tachycardic (114 beats per minute). Physical examination revealed a tremulous male with mild exophthalmos and notable neurological findings of left-sided facial droop and left hemiparesis. National Institutes of Health (NIH) stroke scale was 7/42. On auscultation, there were bilateral coarse breath sounds and irregularly irregular rhythm without apparent murmur. Electrocardiogram showed atrial fibrillation with a rapid ventricular response (RVR) (Figure 1). Chest x-ray indicated emphysematous changes with mild pulmonary edema (Figure 2).

**Figure 3:**
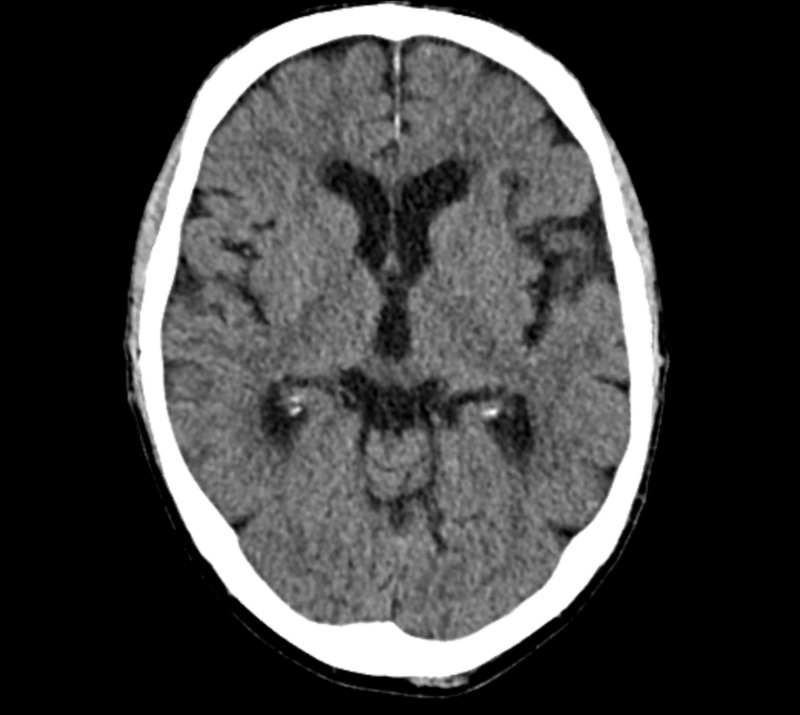
Initial CT of the brain without contrast revealing no acute intracranial abnormality

CT of the head without contrast revealed no signs of acute ischemia or hemorrhage (Figure 3). Thrombolytic therapy was subsequently administered due to concerns for ischemic stroke. Due to atrial fibrillation with RVR and elevated blood pressure, the patient was started on a diltiazem infusion and was admitted to the intensive care unit. Initial laboratory testing revealed white blood cell count of 4.8 K/ul (normal value: 3.9-9.5 K/ul), hemoglobin: 13.4 g/dl (normal value: 12.8-16.6 g/dl), and thrombocytopenia (81 K/ul [normal value: 140-366 K/ul]). There were no noted electrolyte disturbances or alterations in liver or kidney function. 

Within the first two hours of admission, the patient further decompensated with temperature 38.9^o^C, heart rate: 125 beats/minute, tachypnea (44 breaths per minute), and increased hypertension with peak blood pressure of 199/155 mmHg. Flaccidity in the right upper extremity was noted to have developed and the patient became very agitated and aggressive. The patient continued to deteriorate and required eventual endotracheal intubation. CT of the head was repeated, which ruled out intracranial hemorrhage and again revealed no acute intracranial process.

Shortly thereafter, additional laboratory testing resulted and revealed significantly abnormal thyroid function with thyroid-stimulating hormone (TSH): <0.010 uIU/ml (normal value: 0.35-5.5 uIU/ml), free T4: 5.5 ng/dl (normal value: 0.6-1.6 ng/dl), and T3: 348 ng/dl (normal value: 76-181 ng/dl). The patient was diagnosed with hyperthyroidism with thyroid storm. He was started on propylthiouracil (PTU) in addition to potassium iodide and glucocorticoids. Due to difficulty controlling blood pressure and heart rate, diltiazem was discontinued and esmolol infusion was initiated. The patient converted from atrial fibrillation to normal sinus rhythm within 24 hours; however, remained persistently tachycardic.

**Figure 4:**
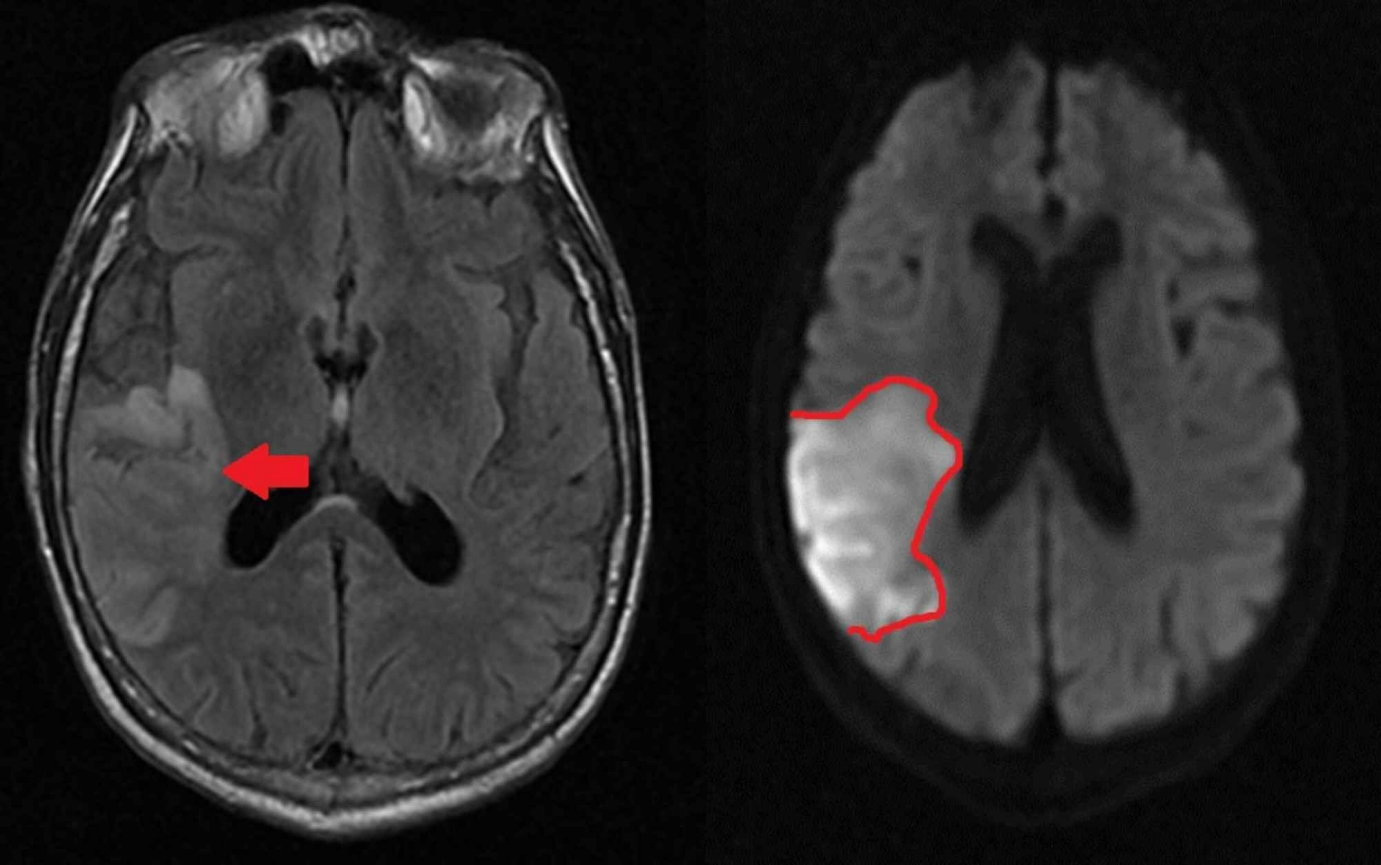
Large right middle cerebral artery region infarct visualized with MRI *Left: *Axial view MRI with fluid-attenuated inversion recovery (FLAIR). Area of infarct is indicated by an arrow.* Right:* MRI with diffusion-weighted imaging (DWI), infarcted area has been outlined

MRI of the brain showed large sub-acute infarct in the posterior right middle cerebral artery distribution (Figure 4). Transthoracic echocardiogram revealed an ejection fraction of 55% to 60% and moderately dilated left atrium; no thrombus formation was visualized. The patient was started on atorvastatin and clopidogrel for secondary stroke prevention. Esmolol infusion was transitioned to oral propranolol as blood pressure and heart rate improved. Given atrial fibrillation, apixaban was initiated two weeks after initial stroke during which time clopidogrel was discontinued.

Thyroid-stimulating immunoglobulins came back elevated at 381 (normal value: >140% baseline), confirming the diagnosis of Grave’s disease. Post extubation, the patient underwent total thyroidectomy. PTU was continued for 48 hours post thyroidectomy, steroids were tapered, and levothyroxine was initiated.

The patient’s symptoms improved significantly and he was discharged to an inpatient rehabilitation center on levothyroxine, atorvastatin, apixaban, and metoprolol tartrate. At a four-week follow-up visit with an endocrinologist, the patient denied any symptoms of continued thyroid dysfunction. Thyroid function tests improved to TSH: 0.199 uIU/ml and free T4: 0.5 ng/dl. Neurological status was improved with noted mild dysarthria and continued left-sided upper extremity weakness. 

## Discussion

Thyroid storm is a rare medical emergency resulting in extreme excess of circulating thyroid hormones in a state known as thyrotoxicosis [8]. Hyperthyroidism may result from increased production of thyroid hormones or from exogenous thyroid hormones [8-11]. Events that precipitate thyroid storm include infection, surgery, emotional stress, iodine load, medication non-compliance, and other acute medical illnesses [2-5,8,11].

Timely diagnosis of thyroid storm is challenging and is often missed due to non-specific symptoms, which reflect an increased metabolic state (Table 1). The diagnosis of thyroid storm is based on clinical findings and symptoms of hyperthyroidism accompanied by manifestations of multi-organ failure. Diagnosis is further supported by thyroid function tests with low TSH and elevated free T3 and T4 levels [1,3,7,8,11]. 

**Table 1:** Symptoms of thyrotoxicosis

Thyrotoxicosis symptoms	
Generalized	Fatigue/generalized weakness
	Weight changes
	Increased sweating
	Heat intolerance
Neurological	Agitation/irritability
	Tremor
	Seizures
Cardiac	Tachycardia/palpitations
Gastrointestinal	Appetite changes
	Abdominal pain
	Diarrhea
	Nausea/vomiting

Ischemic stroke presenting with thyroid storm is a rare occurrence. Ischemic stroke can be both the cause and the effect of the thyroid storm [8,12]. Ischemic stroke as a result of thyroid storm has two possible mechanisms: atrial fibrillation and hypercoagulable state. Atrial fibrillation is a well-known risk factor for stroke and occurs in 10% to 35% of thyrotoxicosis patients with increased incidence in patients over the age of 60 [6,8,11,13]. In contrast to this, a hypercoagulable state is produced during thyrotoxicosis due to a shortened activated partial thromboplastin time, increased fibrinogen levels, and increased factor VIII and factor X activity which predispose a patient to stroke regardless of the heart rhythm [2,11,13,14]. Despite the propensity for hypercoagulability, evaluation of thyrotoxicosis is not currently part of the recommended workup for ischemic strokes as it is for atrial fibrillation [2,7,15]. In a large retrospective study, Petersen and Hanson demonstrated that patients with thyrotoxicosis and atrial fibrillation are at no increased risk of thromboembolic events compared with aged-matched patients also with atrial fibrillation [9].

Once a thyroid storm is established as a diagnosis, it is imperative to identify and treat the underlying cause. In the case of ischemic stroke, routine stroke management is recommended with tPA (tissue plasminogen activator) (if within the time limits for administration), blood pressure control, and frequent neurological monitoring. Due to increased peripheral conversion of T4 to T3, aspirin should be avoided, if possible, in cases of acute thyrotoxicosis [5,11]. Clopidogrel may be considered as an alternative [5,11]. Routine anticoagulation of thyrotoxicosis patients, in the absence of atrial fibrillation, is not currently recommended and should be based on risk factors such as age and clinical judgment [7,15]. Anticoagulation is recommended in the presence of atrial fibrillation and is guided by the use of the CHADS-VASc scoring system, which does not include hyperthyroidism as a risk factor [2,7,13,15]. 

Of importance, if anticoagulation with warfarin is considered, it should be initiated at lower doses due to reduced levels of vitamin K associated clotting factors in thyrotoxicosis patients [9,14,15]. Until recently, the use of novel oral anticoagulants (NOACs) in thyrotoxicosis has been poorly elucidated in the literature. Prior publications have expressed concerns that due to changes in the coagulation pathway, bleeding may occur at increased rates in these patients [15,16]. Recently, however, Goldstein et al. published a secondary analysis of the ARISTOTLE trial (Apixaban for Reduction in Stroke and Other Thromboembolic Events in Atrial Fibrillation) specifically observing clinical characteristics of patients with atrial fibrillation and those with and without thyroid disease treated with apixaban [13]. This study indicated that apixaban was superior to warfarin irrespective of thyroid disease history [13]. Unfortunately, this study cannot be generalized to patients with uncontrolled hyperthyroidism in the case of thyroid storm. 

After the treatment of inciting illness, successful treatment of thyroid storm is dependent on early reduction of thyroid hormone production and decreased extrathyroidal conversion of T4 to T3. PTU and methimazole are the mainstays in therapy and act to decrease follicular growth and reduce thyroid peroxidase synthesis, thereby decreasing the synthesis of T4 and T3 [2,3,11,17]. In thyroid storm, PTU is typically favored over methimazole due to its ability to additionally reduce peripheral conversion of T4 to T3 [2,8]. In patients such as those with acute stroke who are unable to receive these medications enterally, both PTU and methimazole can be prepared and administered via rectal suppository or enema [2,8]. This route provides slightly diminished bioavailability when compared to oral formulations; however, intravenous methimazole as an alternative is available [2]. 

Non-radioactive iodide may also be considered as it can decrease thyroid hormone production by causing plasma iodide levels to reach a threshold wherein iodide is unable to bind to thyroglobulin in the thyroid gland [2]. It is imperative that if iodide is used that it be administered at a minimum of 30 minutes after any thiouracil as co-administration may increase thyroid hormone production, thereby worsening thyroid storm [2,8,11,17]. Additional reduction of extra-thyroidal conversion of T4 to T3 can be aided by glucocorticoids, which additionally act in the prevention of adrenal insufficiency that occurs at increased rates in thyroid storm patients [2]. In refractory cases, circulating thyroid hormones can be further decreased with the use of cholestyramine and plasmapheresis [2,11].

Beta antagonists play a crucial role in therapy by slowing the heart rate and decreasing oxygen demand as well as decreasing peripheral conversion of T4 to T3 [2,3,11,17]. Moreover, there is evidence that beta antagonists can reduce adrenergic symptoms, which can minimize fevers, decrease convulsions, and improve psychotic symptoms [11]. When using beta antagonists, it is important to note that their half-life is shortened by circulating thyroid hormones [12]. Among the beta antagonists, propranolol is the most commonly utilized as it is non-selective and easy to administer both orally or intravenously [2,8,11]. In situations that require rapid onset of action, IV propranolol bolus or IV esmolol first as a bolus and then continuous infusion can be considered as both have similar times to onset of action [2,3]. In patients presenting with cardiomyopathy or those with valvular disease, esmolol is the preferred agent over propranolol as it may induce cardiovascular collapse in these scenarios [3,15,17]. Due to esmolol’s short half-life, it can be rapidly discontinued if hypotension or adverse symptoms develop [3,8,17]. Moreover, esmolol is more beta-1 selective when compared to propranolol, making it safer for patients who may be predisposed to bronchospasm [3]. For similar reasons, in thyroid storm with hypertensive emergency and/or acute stroke requiring strict blood pressure control, esmolol may be the preferred agent. 

The majority of patients with thyrotoxicosis can be managed pharmacologically. However, in some situations, thyroidectomy or thyroid ablation may need to be considered. In emergent cases, thyroidectomy is considered only after all attempts at euthyroidism through medical management have failed as surgery can precipitate additional thyroid storm [11,17]. In patients still requiring steroids and beta-blockers at the time of surgery, it is recommended that these medications be slowly weaned over several weeks post-operatively [8,17]. In those who are more stable and are able to be managed through medications alone, thyroid ablation is considered only when non-compliance is problematic in management. In those patients who remain on oral therapy after discharge, methimazole is preferred over PTU due to less frequent dosing and fewer side effects [8,11]. PTU is, however, the agent of choice in the first trimester of pregnancy due to teratogenicity of methimazole [8,11]. 

## Conclusions

In the case discussed, initial concerns for thyrotoxicosis were peaked primarily by physical exam and continued difficulty with controlling fevers, heart rate, and blood pressure. Correctly diagnosing thyroid storm in patients with stroke and without a known diagnosis of hyperthyroidism remains challenging due to its clinical overlap with other medical conditions. In the case of acute stroke with atrial fibrillation, it is imperative that thyrotoxicosis always be considered as immediate initiation of medical therapy to control thyroid storm can limit the morbidity and mortality. 
